# EMX2OS plays a prognosis-associated enhancer RNA role in gastric cancer

**DOI:** 10.1097/MD.0000000000027535

**Published:** 2021-10-15

**Authors:** Ge-Xin Liu, Yu-Zhen Tan, Guo-Chao He, Qin-Lin Zhang, Pan Liu

**Affiliations:** a Department of Emergency, Zhuzhou Central Hospital, Zhuzhou, China; b Department of Neurology, Zhuzhou Central Hospital, Zhuzhou, China.

**Keywords:** EMX2OS, enhancer RNAs, gastric cancer, survival

## Abstract

Enhancer RNAs (eRNAs), a subclass of lncRNAs, are derived from enhancer regions. The function of eRNAs has been reported by many previous studies. However, the role of eRNAs in gastric cancer, especially the prognosis-associated eRNAs, has not been studied yet.

In this study, we have used a novel approach to screened key eRNAs in gastric cancer. Kaplan–Meier correlation analysis and Co-expression analysis were used to find the most significant survival-associated eRNAs. Enrichment analysis is applied to explore the key functions and pathways of screened eRNAs. The correlation and survival analysis are used to evaluate targeted genes in the pan-cancer analysis

A total of 63 prognostic-associated eRNAs in gastric cancer were identified, the top 6 eRNAs were LINC01714, ZNF192P1, AC079760.2, LINC01645, EMX2OS, and AC114489.2. The correlation analysis demonstrated the top 10 screened eRNAs and their targeted genes. The results demonstrated that EMX2OS was ranked as the top eRNA according to the results of the Kaplan–Meier analysis. The correlation analysis demonstrated that eRNA EMX2OS is correlated with age, grade, stage, and cancer status. The pan-cancer analysis demonstrated that EMX2OS was associated with poor survival outcomes in adrenocortical carcinoma, cervical squamous cell carcinoma and endocervical adenocarcinoma, kidney renal clear cell carcinoma, stomach adenocarcinoma, and uveal melanoma.

In this study, survival-related eRNAs were screened and the correlation between survival-related eRNAs and their targeted genes was demonstrated. EMX2OS plays a prognosis-associated eRNA role in gastric cancer, which might be a novel therapeutic target in clinical practice.

## 1 Introduction

Gastric cancer is one of the most common malignant cancers all around the world, with more than 1 million people newly diagnosed with gastric cancer every year.^[1]^ In the past 5 decades, although the incidence and mortality have gradually declined, gastric cancer remains the third leading cause of cancer-associated death. The main cause of gastric cancer attributes to the chronic infection with *H. pylori*.^[2]^ The detection of gastric cancer at an early and curable stage is an effective way to reduce the mortality rate.^[3]^ Besides, *H. pylori* eradication treatment also plays an important role in preventing gastric cancer.^[4]^

Long noncoding RNAs (lncRNAs) has proved to be associated with many kinds of human disease,^[5,6]^ especially cancers.^[7,8]^ Enhancer RNAs (eRNAs), as a subclass of lncRNAs transcribed within gene enhancers, play important roles in lots of biological processes.^[9–11]^ The previous study^[12]^ has reported that eRNAs can interact with transcription factors and RNA polymerase II, so that increase the expression of the corresponding downstream gene. Zhao et al^[13]^ has reported that alterations of androgen receptor-regulated eRNAs could lead to enzalutamide resistance in castration-resistant prostate cancer. To the best of our knowledge, there is no research on gastric-associated eRNAs.

In this study, we have screened a series of prognostic eRNAs and their targeted genes in gastric cancer. Kaplan–Meier correlation analysis and Co-expression analysis were used to find the most significant survival-associated eRNAs. The eRNA EMX2OS was selected and found to be associated with overall survival in gastric cancer patients. Enrichment analysis is applied to explore the key functions and pathways of screened eRNAs. The correlation and survival analysis are used to evaluate targeted genes in the pan-cancer analysis.

## 2 Materials and methods

### 2.1 Data downloading

The gene expression RNAseq profiles, survival data, and clinicopathological characteristics of 33 tumors in the TCGA database were downloaded from the UCSC Xena (https://xena.ucsc.edu/).

### 2.2 Data preparing

The ensemble_IDs in the gene expression RNAseq profiles were transferred into gene symbols. The eRNA_IDs were also transferred into gene symbols. The expression profiles of eRNAs of gastric cancer in the TCGA database were extracted. The survival time and status of patients were merged with the expression profiles of eRNAs of gastric cancer.

### 2.3 Identification of prognostic eRNAs in gastric cancer

We utilized the Kaplan–Meier method to evaluate the prognostic eRNAs in gastric cancer based on the expression level of each eRNAs. The patients were divided into the low-expression group and the high-expression group based on the median expression value.

### 2.4 Validation of the correlation of eRNAs with their targeted genes

The relationship between eRNAs and their targeted genes was evaluated by correlation analysis in gastric cancer. Only those eRNAs with R > 0.4 and *P* < .01 were screened and selected for the following analyses. The plots of correlation analyses were generated. The X-axis represents the expression level of eRNA, and the Y-axis represents the expression of its targeted genes.

### 2.5 Validation of the correlation of the hub eRNA with clinicopathological characteristics

EMX2OS, as one of the most remarkable eRNAs in the Kaplan–Meier analysis and correlation analysis, was selected as the hub eRNA for the following analyses. The correlation of the EMX2OS with clinicopathological characteristics in gastric cancer was performed.

### 2.6 Co-expression analysis of eRNA EMX2OS in gastric cancer

Co-expression analysis was performed in gastric cancer aimed to identify more targeted genes of eRNA EMX2OS. The screened criteria were set as R > 0.4 and *P* < .01.

### 2.7 Enrichment analysis

GO was performed by using the potential targeted genes of eRNA EMX2OS aimed to identify the key functions and pathways in the regulation of gastric cancer.

### 2.8 Validation of the correlation of EMX2OS with its targeted genes in the pan-cancer analysis

The relationship between eRNAs and their targeted genes was evaluated by correlation analysis in pan-cancer.

### 2.9 Validation of the survival analysis of EMX2OS in the pan-cancer analysis

We utilized the Kaplan–Meier method to evaluate the prognostic eRNAs in pan-cancer based on the expression level of each eRNAs. The patients were divided into the low-expression group and the high-expression group based on the median expression value.

## 3 Results

### 3.1 Identification of prognostic eRNAs in gastric cancer

**Figure 1:**
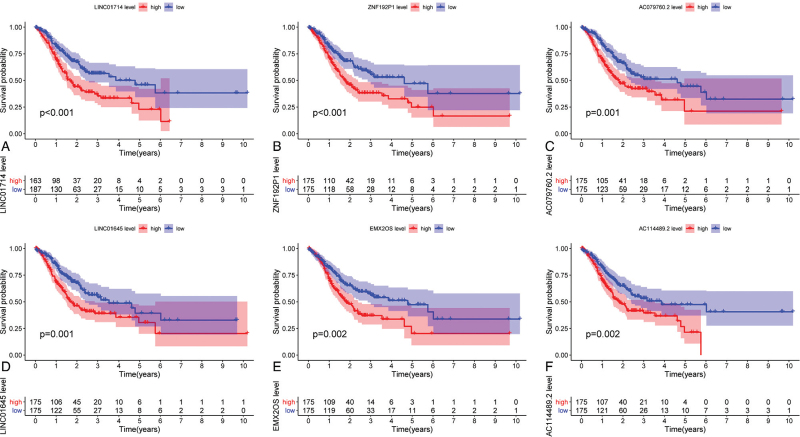
The survival analysis demonstrating the top 6 eRNAs with a *P* value < .01.

A total of 63 prognostic associated eRNAs in gastric cancer were identified. The top 10 eRNAs with *P* < .01 were demonstrated in Table 1. The KM plots of the top 6 eRNAs with *P* < .01 were demonstrated in Figure 1. The results demonstrated that higher expression levels of LINC01714, ZNF192P1, AC079760.2, LINC01645, EMX2OS, and AC114489.2 were significantly associated with the poor survival of gastric cancer patients.

**Table 1:** The list of top 10 survival-related eRNAs screen based on *P*-value.

gene	KM
LINC01714	0.000164
ZNF192P1	0.0006
AC079760.2	0.001022
LINC01645	0.001267
EMX2OS	0.001502
AC114489.2	0.001884
LINC02845	0.001905
AL021937.3	0.00191
AC010457.1	0.002038
HAGLR	0.002276

### 3.2 The correlation of eRNAs with their targeted genes

**Figure 2:**
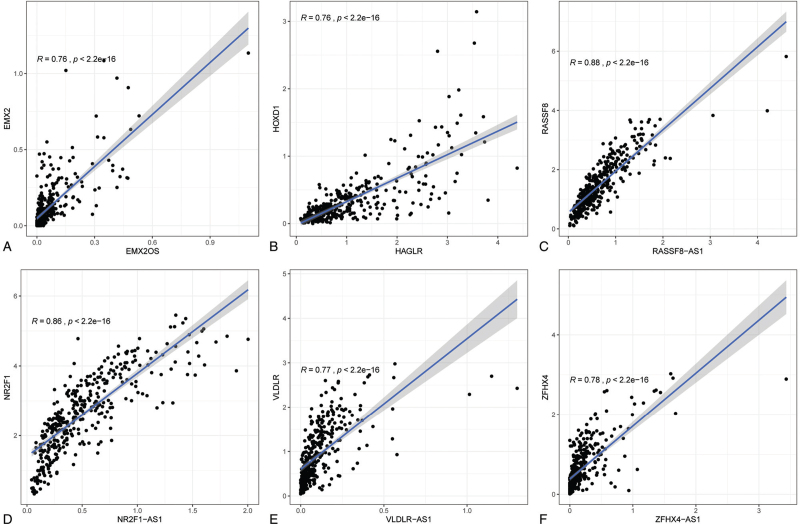
The correlation between the top 6 screened eRNAs and their targeted genes.

The relationship between eRNAs and their targeted genes was evaluated by correlation analysis in gastric cancer. Only those eRNAs with R > 0.6 and *P* < .05 were screened and selected for the following analyses. The correlation between the top 10 screened eRNAs and their targeted genes were presented in Table 2. The correlation between the top 6 screened eRNAs and their targeted genes were presented in Figure 2. The results demonstrated that eRNA EMX2OS with the most significant *P*-value which was calculated by the Kaplan–Meier analysis was selected to perform the following analyses.

**Table 2:** The correlation between the top 10 screened eRNAs and their targeted genes.

eRNA	KM	Target	cor	corPval
EMX2OS	0.001502	EMX2	0.761934	2.31E-72
HAGLR	0.002276	HOXD1	0.758605	0
RASSF8-AS1	0.003017	RASSF8	0.88155	0
NR2F1-AS1	0.005252	NR2F1	0.855387	0
VLDLR-AS1	0.013156	VLDLR	0.774231	4.22E-76
ZFHX4-AS1	0.016982	ZFHX4	0.782502	9.42E-79
LINC02381	0.017657	HOXC4	0.653303	0
AL445426.1	0.024848	WNT2B	0.605051	8.14E-39
AC002451.1	0.035746	PDK4	0.663447	6.40E-49
IGFBP7-AS1	0.041357	IGFBP7	0.778136	2.44E-77

### 3.3 The correlation of eRNA EMX2OS with clinicopathological characteristics

**Figure 3:**
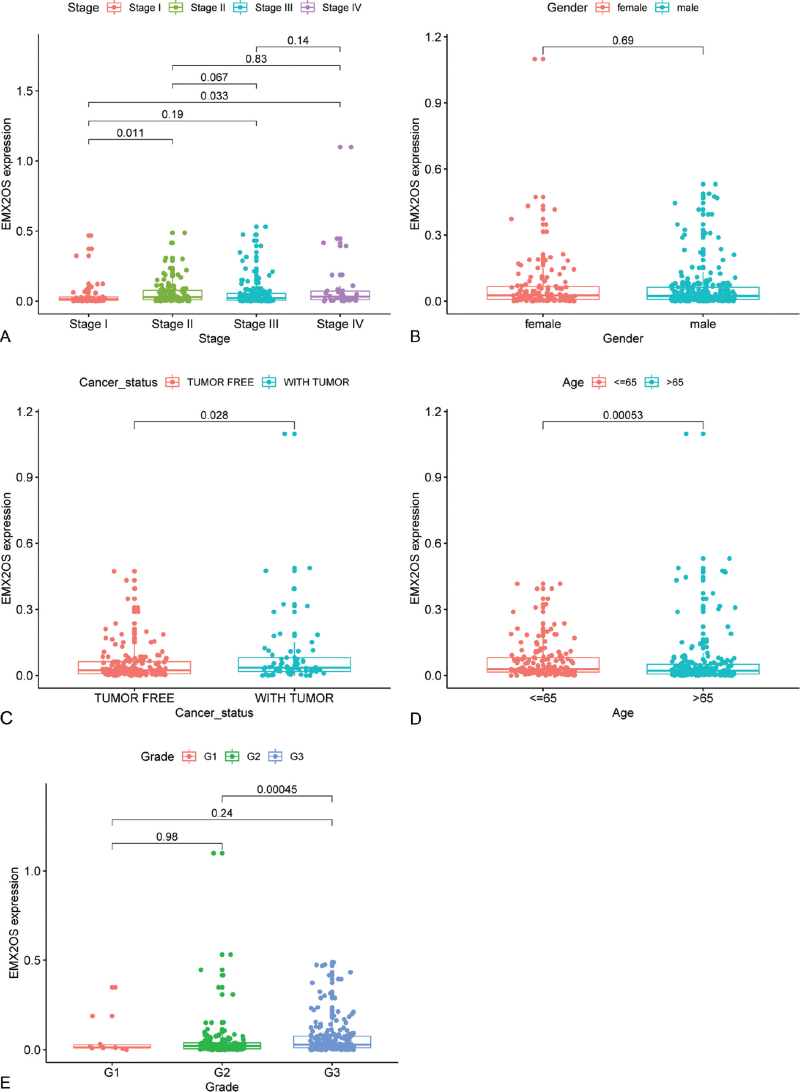
The correlation between eRNA EMX2OS and clinicopathological characteristics.

The clinicopathological characteristics of these patients were demonstrated in Table 3. The clinicopathological characteristics include age, grade, cancer status, and tumor stage. The results demonstrated that eRNA EMX2OS is correlated with the age, grade, stage, and cancer status. eRNA EMX2OS expression in patients with age ≤65 is higher than that in patients with age >65. However, there is no significant difference in terms of gender. In terms of tumor grade, eRNA EMX2OS expression is higher in the G3 grade than those in the G2 grade. For the tumor stage, the results demonstrated that the expression of eRNA EMX2OS is higher in patients with Stage IV than those in Stage I. Also, the expression of eRNA EMX2OS is higher in patients with Stage II than in those with Stage I. The eRNA EMX2OS expression in patients with tumors is higher than those patients with tumor-free (Fig. 3).

**Table 3:** The clinicopathological characteristics of patients.

Covariates	Type	Table_Stat
Age	≤65	164 (43.73%)
	>65	207 (55.2%)
	Unknown	4 (1.07%)
Grade	G1	10 (2.67%)
	G2	137 (36.53%)
	G3	219 (58.4%)
	Unknown	9 (2.4%)
Cancer_status	Tumor free	167 (44.53%)
	Unknown	136 (36.27%)
	With tumor	72 (19.2%)
Gender	Female	134 (35.73%)
	Male	241 (64.27%)
Stage	Stage I	53 (14.13%)
	Stage II	111 (29.6%)
	Stage III	150 (40%)
	Stage IV	38 (10.13%)
	Unknown	23 (6.13%)

### 3.4 Co-expression analysis of eRNA EMX2OS in gastric cancer

Co-expression analysis was performed in gastric cancer aimed to identify more targeted genes of eRNA EMX2OS. The screened criteria were set as R > 0.4 and *P* < .01. A total of 2050 targeted genes were identified finally. The top 10 targeted genes of EXM2OS were demonstrated in Table 4 which ranked according to the correlation value.

**Table 4:** The results of co-expression analysis demonstrating the top 10 targeted genes of EMX2OS.

eRNA	gene	cor	*P*-value
EMX2OS	EMX2	0.762	2.31E-72
EMX2OS	SLITRK2	0.7	1.41E-56
EMX2OS	ST6GAL2	0.691	1.91E-54
EMX2OS	RGS22	0.681	2.43E-52
EMX2OS	RNF165	0.678	8.52E-52
EMX2OS	CYP1B1-AS1	0.669	4.89E-50
EMX2OS	SCN2B	0.668	9.86E-50
EMX2OS	LINC01638	0.665	2.76E-49
EMX2OS	CDO1	0.662	1.26E-48
EMX2OS	LRRC4C	0.659	5.56E-48

### 3.5 Enrichment analysis

**Figure 4:**
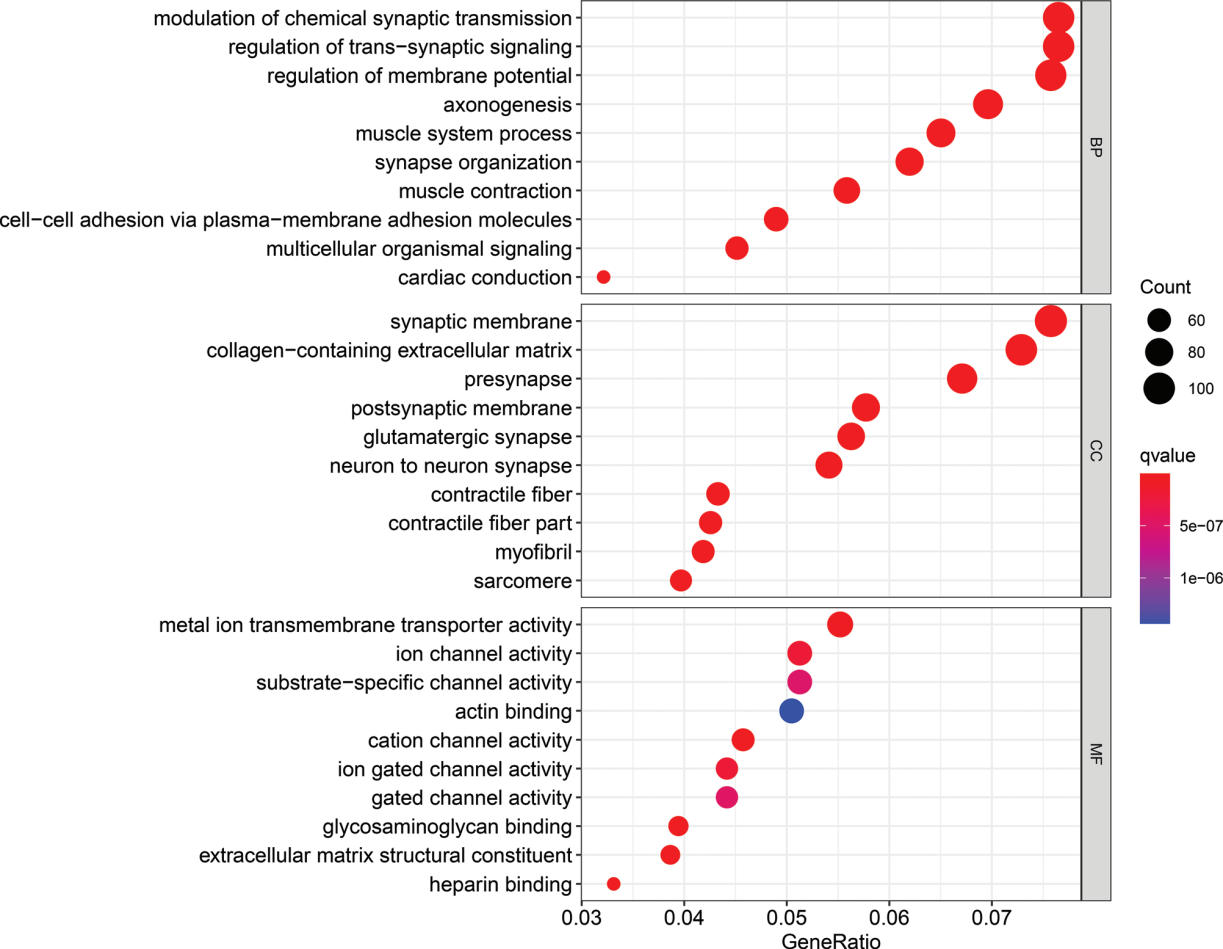
The results of GO analysis were conducted by using the potential targeted genes of eRNA EMX2OS.

GO analyses were performed by using the potential targeted genes of eRNA EMX2OS aimed to identify the key functions in the regulation of gastric cancer. The results were demonstrated in Figure 4. The top 3 terms enriched in BP are modulation of chemical synaptic transmission, regulation of trans-synaptic signaling, regulation of membrane potential. The top 3 terms enriched in CC were synaptic membrane, collagen-containing extracellular matrix, and presynapse. The top 3 terms enriched in MF were metal ion transmembrane transporter activity, ion channel activity, and substrate-specific channel activity.

### 3.6 The correlation of eRNA EMX2OS with its targeted genes and survival analyses in pan-cancer

**Figure 5:**
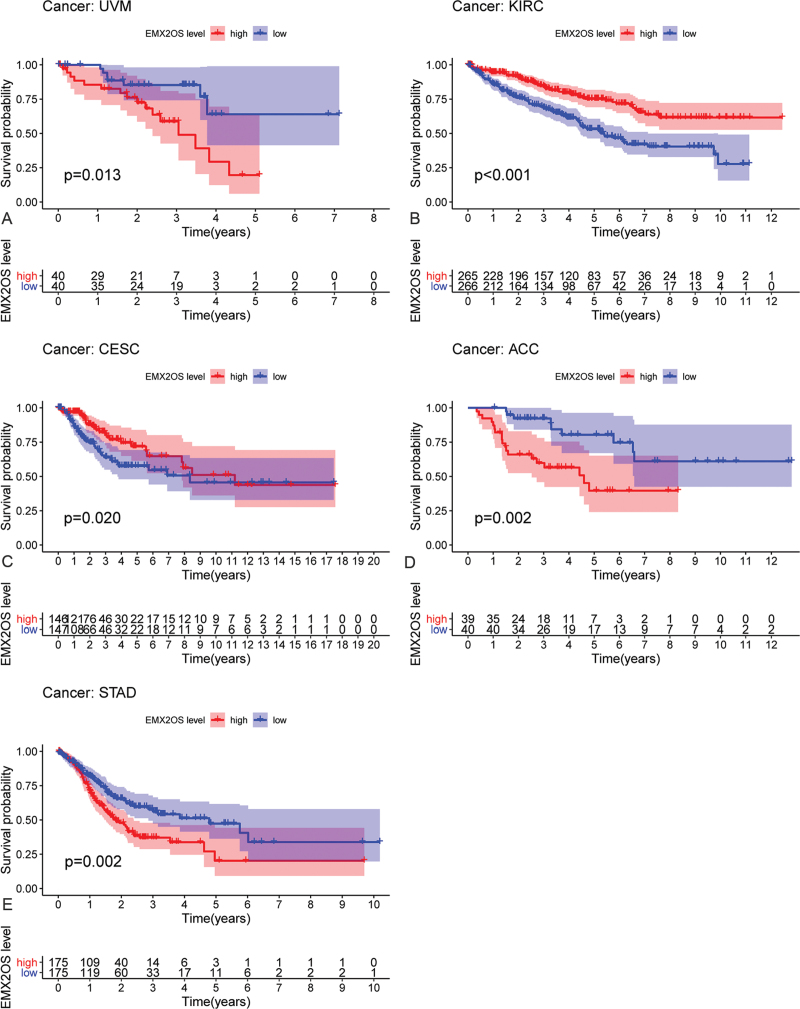
Survival analyses of EMX2OS stratified by the relative expression of EMX2OS in pan-cancer, including (D) adrenocortical carcinoma, (C) cervical squamous cell carcinoma, and endocervical adenocarcinoma, (B) kidney renal clear cell carcinoma, (E) stomach adenocarcinoma, and (A) uveal melanoma.

**Figure 6:**
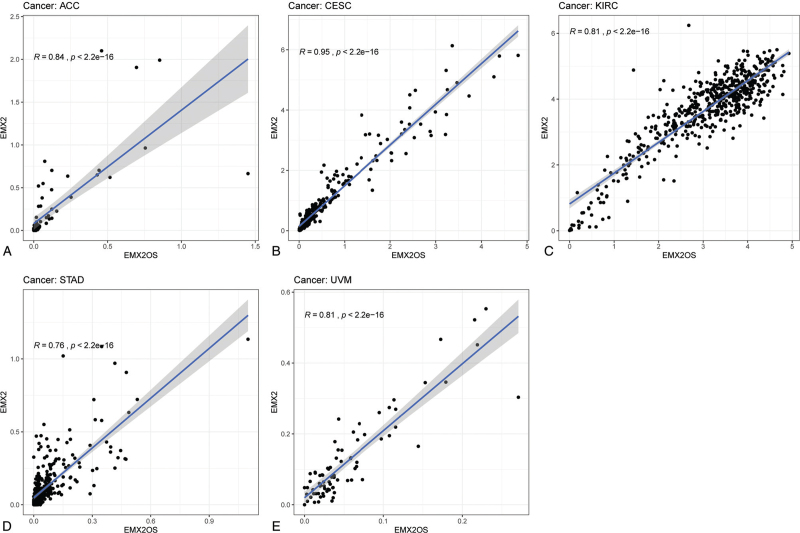
The correlation between eRNA EMX2OS and its targeted gene EMX2 in pan-cancer, including (A) adrenocortical carcinoma, (B) cervical squamous cell carcinoma, and endocervical adenocarcinoma, (C) kidney renal clear cell carcinoma, (D) stomach adenocarcinoma, and (E) uveal melanoma.

The correlation between eRNA EMX2OS and its targeted genes in pan-cancer was calculated. We utilized the Kaplan–Meier method to evaluate the prognostic eRNAs in pan-cancer based on the expression level of each eRNAs. The patients were divided into the low-expression group and the high-expression group based on the median expression value. Finally, we identified that eRNA EMX2OS also plays important roles in 5 tumors, including adrenocortical carcinoma (Fig. 5D, Fig. 6A), cervical squamous cell carcinoma and endocervical adenocarcinoma (Fig. 5C, Fig. 6B), kidney renal clear cell carcinoma (Fig. 5B, Fig. 6C), stomach adenocarcinoma (Fig. 5E, Fig. 6D), and uveal melanoma (Fig. 5A, Fig. 6E). These results mentioned above reflected that eRNA EMX2OS may play critical roles in many types of tumors and may have the chance to be regarded as a novel treatment target in the future.

## 4 Discussion

eRNAs, as a specific subclass of lncRNAs, are derived from gene enhancer regions. eRNAs play an important role in regulating the transcription of the corresponding genes and tumorigenesis.^[14]^ The previous study^[15]^ has reported that eRNAs play an important role in increasing the rate of the transcription of genes. As the evidence began to accumulate, the importance of the role of the eRNAs in tumor development and treatment has been reported by many researchers. For instance, Zhang et al^[9]^ demonstrated that eRNAs can be regarded as a useful tool for eRNA-targeted therapy in cancer. Also, Wang et al^[14]^ showed that WAKMAR2 might be a novel target gene in regulating the breast cancer microenvironment and may function by influencing the expression of immune-related genes. However, the specific role of eRNAs in cell fate determination has not been completely elucidated yet. In this study, we performed a series of assays to identify the prognosis-related eRNAs in gastric cancer. To illustrate the network of eRNAs in gastric cancer, we identified a subset of eRNAs that are significantly associated with the overall survival of gastric cancer. Then, we have identified a subset of eRNAs’ potential target genes. To the best of our knowledge, this is the first study to explore the influence of eRNAs in gastric cancer.

In this study, the top key eRNA candidate in gastric cancer is EMX2OS. EMX2OS, as a lncRNA, has been reported to play an important role in many cancers. Duan et al^[16]^ reported that lncRNA EMX2OS can induce proliferation, invasion, and sphere formation of ovarian cancer cells via regulation of the miR-654-3p/AKT3/PD-L1 Axis. Also, Gu et al^[17]^ demonstrated that the downregulation of lncRNA EMX2OS can predict shorter recurrence-free survival of thyroid cancer. Plus, a previous study^[18]^ reported that lncRNA EMX2OS was significantly upregulated in gastric cancer tissues compared with normal tissues, which is consistent with our results. However, the effects and mechanisms of EMX2OS in gastric cancer remain largely unknown.

EMX2OS is located in the enhancer region of the EMX2 gene. EMX2, a protein-coding gene, was reported to be a significant role in many kinds of tumors. Aykut et al^[19]^ demonstrated that EMX2 is down-regulated in colorectal cancer and can be a prognostic marker for disease-free and overall survival of colorectal cancer. Also, Falcone et al^[20]^ demonstrated that EMX2 could be treated as a novel promising tool for the treatment of glioblastoma and prevention of its recurrences.

The results of this study have shown a strong correlation between the expression of EMX2OS and EMX2. Also, EMX2OS has shown the most significant impact on overall survival in gastric cancer among all identified eRNAs. Plus, we have identified co-expression genes of EMX2OS in addition to its targeted gene EMX2. Finally, we have found a total of 2050 genes with a significant expression correlation with EMX2OS. Some studies indicated that the primary function of eRNA was performed in cis, however, several studies stated that eRNAs could also regulate the expression of genes in trans.^[21]^ To determine the function of screened genes with significant expression correlation with EMX2OS, we performed gene ontology enrichment analysis.

Recently, some researchers have reported that the diet, treatment, and heterogeneity of tumors play important roles in affecting the expression of eRNAs. For instance, Klein et al.^[22]^ demonstrated the effect of early-life undernutrition on the induction of the eRNA remodeling in mice liver. They stated that the expression of metabolism-associated genes was regulated by the enhancer activity in early life. In terms of the treatment, Xie et al^[23]^ demonstrated that knockdown enhancer-RNA-P53-bound enhancer regions 2 could reverse the nutlin-3-induced cytotoxic effect in TP53-WT cell lines. Chen et al^[24]^ showed that the eRNAs could affect the cancer phenotypes by regulating the intra-tumoral heterogeneity. In summary, the diet, treatment, and heterogeneity of tumors could affect the function of eRNAs.

There are some limitations to this study. Firstly, the number of cases in this study is too small, for not further data could be downloaded from other databases. Secondly, no external data and historic comparisons were used to make the validation of our conclusion. Further study should be made to validate our conclusion, including external data and historic validations. Third, the effect of diet, treatment, and heterogeneity of tumors on the expression of eRNAs has not been deeply explored in this study. However, we have tried our best to make a literature review in the discussion part of this study.

To conclude, we have identified key eRNAs in gastric cancer. The impact of these eRNAs on overall survival was also demonstrated. The results suggested that eRNA EMX2OS is a prognosis-related gene for gastric cancer. eRNA EMX2OS may be a therapeutic target for patients with gastric cancer.

## Acknowledgments

Not applicable.

## Author contributions

**Conceptualization:** GeXin Liu, Pan Liu.

**Data curation:** GeXin Liu, GuoChao He.

**Formal analysis:** GuoChao He.

**Methodology:** Yu-Zhen Tan.

**Software:** Yu-Zhen Tan.

**Supervision:** QinLin Zhang, Pan Liu.

**Visualization:** QinLin Zhang.

**Writing – original draft:** GeXin Liu, Yu-Zhen Tan.

**Writing – review & editing:** Pan Liu.
